# Supplementary material for: Causes and consequences of sex-chromosome turnovers in Diptera
Source: Evol Lett. 2026 Mar 12;10(3):253–68. doi: 10.1093/evlett/qrag003 (PMC13231274; doi:10.1093/evlett/qrag003)
Supplement: qrag003_Supplemental_Files [file qrag003_supplemental_files.zip › Supplementary_Datasets.zip!/Supplementary_Datasets/Dataset_2_Graphical_representation_outgroups/Sciaridae_Bradysia_coprophila_chromosomes.pdf]

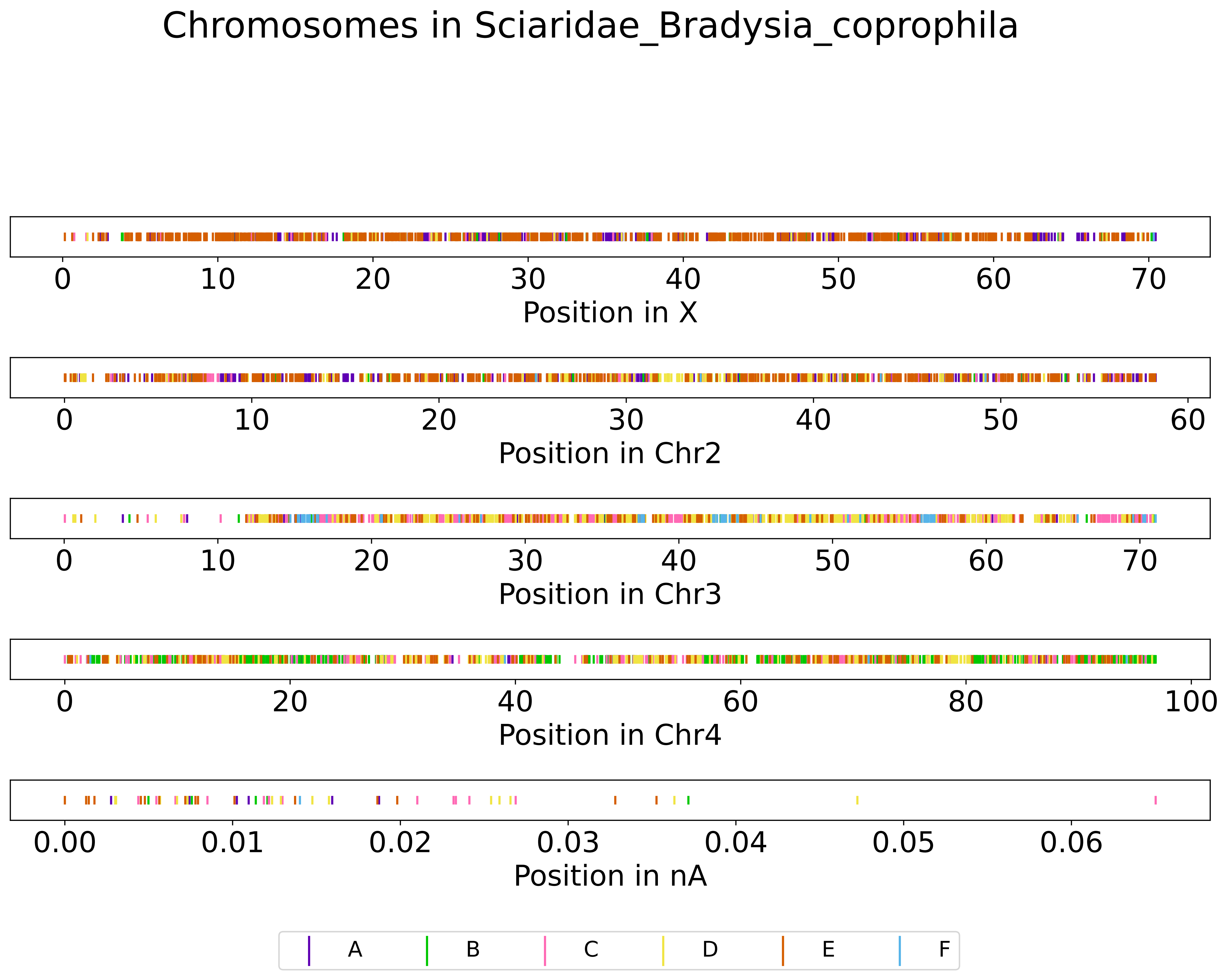
Chromosomes in Sciaridae_Bradysia_coprophila
0 10 20 30 40 50 60 70
Position in X
0 10 20 30 40 50 60
Position in Chr2
0 10 20 30 40 50 60 70
Position in Chr3
0 20 40 60 80 100
Position in Chr4
0.00 0.01 0.02 0.03 0.04 0.05 0.06
Position in nA
A
B
C
D
E
F
